# Coexistence of Two Rare Sarcomas: Primary Leiomyosarcoma of Bone and Epithelioid Hemangioendothelioma of the Liver

**DOI:** 10.1155/2008/416085

**Published:** 2008-03-12

**Authors:** E. Gonzalez-Billalabeitia, M. Quintela-Fandino, I. Alemany, G. López-Alonso, A. Ruiz-Ollero, F. Martinez-Tello, R. Hitt

**Affiliations:** ^1^The Division of Medical Oncology, “12 de Octubre” University Hospital, Madrid 28041, Spain; ^2^The Division of Pathology, “12 de Octubre” University Hospital, Madrid 28041, Spain; ^3^The Division of Radiology, Hospital Clínico de Madrid, Madrid 28040, Spain

## Abstract

A 33-year-old woman sought medical attention for a painful swelling of the left ankle. Plain radiographs revealed an osteolytic lesion involving the left distal tibia. An excisional biopsy provided the diagnosis of leiomyosarcoma in the tibia. A staging work-up was performed and an abdominal CT showed 4 liver hypodense lesions in both lobes with peripheral contrast enhancement. A liver biopsy confirmed the diagnosis of epithelioid hemangioendothelioma of the liver. No association between these two entities has been described before. This case introduces the importance of the pathological confirmation of apparent metastatic lesions in low grade sarcomas and provides a review of the literature of both tumours.

## 1. CASE REPORT

Primary leiomyosarcoma of the tibia and epithelioid hemangioendothelioma of the liver are rare soft tissue sarcomas with few cases reported. No association between these two clinical entities has been described before. This case introduces the importance of the pathological confirmation of apparent metastatic lesions.

**Figure 1:**
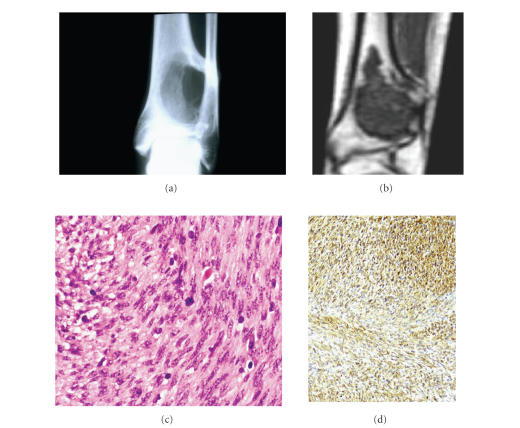
(a) Conventional radiograph reveals an osteolytic lesion in metadiaphysis of the left distal tibia with cortical destruction. (b) T1-Weightened Magnetic Resonance Image shows a well-defined low density intramedullary location with cortical breakthrough. (c) Interlacing bundles of spindle cells with enlarged shaped atypical nuclei and eosinophilic cytoplasm (H&E, x40). (d) Tumor cells are immunostained by the antibody against Actin HHF-35.

**Figure 2:**
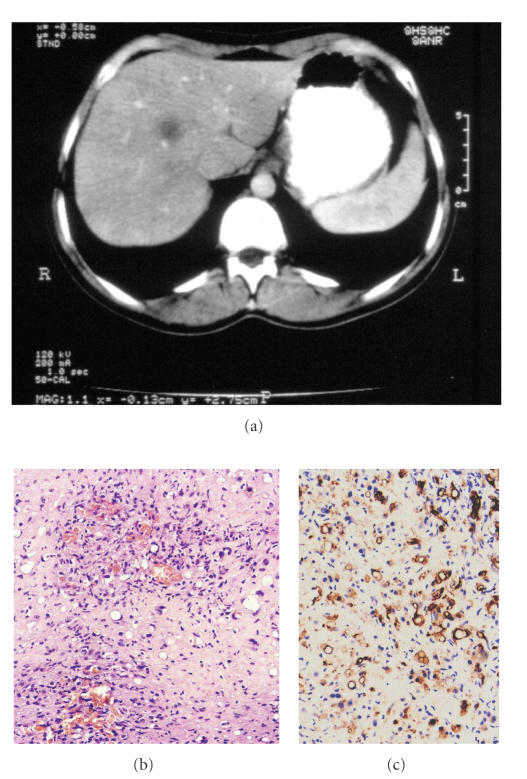
(a) Abdominal computed tomography showing an hypodense lesion in the liver. (b) Tumor composed of fibrous stroma with myxohyaline areas containing epithelioid cells with intracellular vacuoles (H&E, x20). (c) Tumor cells are immunostained with antibody to CD34.

A 33-year-old woman sought medical attention for a two-month progressive painful swelling of the left ankle. Plain radiographs revealed a 5 cm osteolytic lesion involving the left distal tibia metadiaphysis with cortical destruction and no periosteal reaction (Figure 1). An excisional biopsy was performed and the surgical defect was primarily reconstructed with an autogenous free iliac crest bone graft. Light microscopy revealed a tumor constituted mainly of spindle cells with an interlaced pattern, enlarged shaped atypical nuclei and eosinophilic cytoplasm. Necrotic areas and collagen bundles were apparent. No osteoid tissue was observed. Immunohistochemical examination showed positive staining for smooth muscle actin (HHF-35 and IA-4), desmin, and vimentin; and negative staining for cytokeratins (AE1 and AE3), S-100 protein, and CD31. The pathological diagnosis was consistent with leiomyosarcoma in the tibia. An abdominal CT showed 4 liver hypodense lesions in both lobes, the longest 3 cm in length with peripheral contrast enhancement (Figure 2). An exhaustive extension study that included a gynecological examination, bone scan, and thoracic CT observed no other lesions. A liver MRI confirmed the existence of four lesions, with low signal intensity on T1-weightened images and high signal intensity on T2-weightened images, and gadolinium enhancement. A positron emission tomography was performed and 2 enhancements were observed in hepatic segment VII and VIII, with a standard uptake value (SUV) of 2.8.

The woman sought medical attention at our hospital. Due to the atypical clinical presentation and course, a liver biopsy was performed. Macroscopically there was a white, firm lesion. Histologically the tumor was composed of a fibrous stroma, with myxohyaline areas, as well as epithelioid and dendritic cells containing intracellular vacuoles. Immunohistochemistry revealed positive staining for endothelial markers CD31 and CD34, cytokeratin (AE1 and AE3), and vimentin. Smooth muscle actin (HHF and 1A4), desmin, and S-100 protein staining were negative. The pathological diagnosis was consistent with epithelioid hemangioendothelioma. Both pathological samples were compared by two different pathologists confirming the coexistence of two distinct pathological entities. Chemotherapy was no longer continued, and a watch and wait policy was assumed. The patient is four years later under close follow-up with no evidence of progression of the epithelioid hemangioendothelioma of the liver and no signs of recurrence of the leiomyosarcoma in the tibia.

Primary leiomyosarcoma of the bone is a very rare tumor first reported by Evans and Sanerkin [1]. Many cases have been reported since then [2] most in facial bones with extrafacial cases accounting for less than a hundred. The most frequently involved sites are the femur and the tibia. Median age of presentation is about 40 years. The typical clinical presentation is a progressive painful swelling with a palpable mass. Time to diagnosis usually exceeds 6 months. Pathological characteristics of the leiomyosarcoma of bone are similar to leiomyosarcoma in other locations. Immunohistochemistry showing smooth muscle antigens strongly suggest the diagnosis. Leiomyosarcoma usually arises in the uterus, gastrointestinal tract, or soft tissue. All these locations should be carefully excluded to assess primary origin. Despite primary lesions tending to be higher, purely osteolytic and with a distal predomination, differentiation from secondary lesions may be difficult. Primary leiomyosarcoma of the bone can metastasize to the lung, followed by lumbar spine and liver. The best treatment is to resect the whole tumor. Chemotherapy and radiotherapy are of unknown efficacy.

Epithelioid hemangioendothelioma is an infrequent soft tissue tumor of vascular origin. It was first described in the lung by Dail and Liebow [3]. Initially, it was known as intravascular bronchioloalveolar tumor (IVBAT). Ultrastructural examination and the immunochemical expression of factor VIII-related antigen demonstrated the vascular nature of this tumor. Weiss and Enzinger [4] described a group of soft tissue tumors of endothelial origin with a clinical course intermediate between hemangioma and angiosarcoma, which they called epithelioid hemangioendothelioma (EHE). Less than three hundred cases involving the liver are available in the literature. The larger clinicopathologic study involving 137 cases was published by Makhlouf et al. [5]. Median age of diagnosis is 46 years, with a slight predominance in females. The clinical course ranges from asymptomatic diagnosis to liver failure. The most frequent presentation is a nonspecific pain in the right upper quadrant. Pathologic diagnosis is based on the demonstration of the vascular nature of the tumor. Histologically it is comprised of a fibrous stroma with myxohyaline areas with dendritic and epithelioid cells often containing intracellular vacuoles. Immunohistochemistry shows expression of endothelial antigens such as factor VIII-related antigen, CD34, or CD31. High cellularity more than mitotic count predicts an unfavorable prognosis. There is an apparent dissociation between the biologic characteristics of this tumor and the clinical behavior. The clinical course is unpredictable with prolonged survival described up to 27 years without treatment and more than 43% of patients live longer than 5 years. Treatment options are limited by the rarity of the tumor. Complete surgical excision should be performed if possible. Despite being believed to be resistant to chemotherapy and radiotherapy, responses to doxorubicin, thalidomide, and interferon have been reported. Partial spontaneous regression has also been described. Orthotopic liver transplantation is the only option in patients with progressive liver failure. To date no association between these tumors has been described.
